# miR-146a-3p as a potential novel therapeutic by targeting MBD2 to mediate Th17 differentiation in Th17 predominant neutrophilic severe asthma

**DOI:** 10.1007/s10238-023-01033-0

**Published:** 2023-03-24

**Authors:** Wentao Duan, Jin Huang, Binaya Wasti, Zhifeng Chen, Yu Yuan, Yi He, Danhong Li, Jingsi Jia, Shaokun Liu, Yi Liu, Libing Ma, Qingping Zeng, Liming zhu, Jianmin Li, Xiufeng Zhang, Xudong Xiang

**Affiliations:** 1 Department of Respiratory and Critical Care Medicine, The Second Xiangya Hospital, Central South University, Changsha, 410011 China; 2 Changsha Social Work College, Changsha, 410004 China; 3 Department of Emergency, The Second Xiangya Hospital, Central South University, 139 Middle RenminRoad, 410011 Changsha, China; 4 Department of Respiratory and Critical Care Medicine, Zhuzhou City Central Hospital, Zhuzhou, 412007 China; 5 Department of Respiratory and Critical Care Medicine, The Affiliated Hospital of Guilin Medical College, Guilin, 541001 China; 6 Department of Respiratory and Critical Care Medicine, Longshan County People’s Hospital, Longshan, 416800 China; 7 Department of Respiratory and Critical Care Medicine, Hunan Provincial People’s Hospital (The First-Affiliated Hospital of Hunan Normal University), Guhan Road No. 89, Changsha, 410016 China; 8 Department of Respiratory Medicine, The Second Affiliated Hospital of Hainan Medical College University, 570000 Haikou, China

**Keywords:** Severe asthma, miR-146a-3p, MBD2, Th17

## Abstract

Th17 (T-helper 17) cells subtype of non-T2 (non-type 2) asthma is related to neutrophilic infiltration and resistance to inhaled corticosteroids (ICS), so is also known as severe asthma. Methyl-CpG binding domain protein 2 (MBD2) regulates the differentiation of the Th17 cells, tending to show a therapeutic target in severe asthma. miR-146a-3p is associated with anti-inflammatory characteristics and immunity. Moreover, bioinformatic analysis showed that MBD2 may be a target gene of miR-146a-3p. However, the role of miR-146a-3p in the differentiation of Th17 cells via MBD2 in severe asthma remains unknown. Here, we aimed to explore how miR-146a-3p interacts with MBD2 and affects the differentiation of Th17 cells in severe asthma. First, we recruited 30 eligible healthy people and 30 patients with severe asthma to detect the expression of miR-146a-3p in peripheral blood mononuclear cells (PBMCs) by qRT-PCR. Then, we established a HDM/LPS (house dust mite/lipopolysaccharide) exposure model of bronchial epithelial cells (BECs) to evaluate the expression of miR-146a-3p, the interaction between miR-146a-3p and MBD2 using western blot and luciferase reporter analysis and the effect of miR-146a-3p regulated Th17 cells differentiation by flow cytometry in BECs in vitro. Finally, we constructed a mouse model of Th17 predominant neutrophilic severe asthma to assess the therapeutic potential of miR-146a-3p in severe asthma and the effect of miR-146a-3p regulated Th17 cells differentiation via MBD2 in vivo. Decreased miR-146a-3p expression was noted in severe asthma patients, in the BECs and in the animal severe asthma models. Moreover, we demonstrated that miR-146a-3p suppressed Th17 cells differentiation by targeting the MBD2. miR-146a-3p overexpression significantly reduced airway hyperresponsiveness, airway inflammation and airway mucus secretion, while also inhibiting Th17 cells response in vivo, which relieved severe asthma. By targeting MBD2 to suppress Th17 cells differentiation, miR-146a-3p provides a potential novel therapeutic for Th17 predominant neutrophilic severe asthma.

**Supplementary Information:**

The online version contains supplementary material available at 10.1007/s10238-023-01033-0.

## Introduction

Asthma is a heterogeneous disease with variable clinical presentations (phenotypes) and distinct pathological features (endotypes) [1]. According to inflammatory and immune mechanisms, asthma is divided into two major endotypes: “T-helper 2 (Th2)/type2(T2)asthma(eosinophilic)” and “non-T-helper 2 (Th2)/type 2 (T2)asthma (non-eosinophilic)”, which is the most well-established classification of severe asthma endotypes [2]. The airway of T2 asthma is mainly infiltrated by eosinophils and has a good response to inhaled corticosteroid, so it is also called “corticosteroid-sensitive asthma”. However, research using airway epithelial transcriptome analysis has shown that T2-biased airway inflammation is observed in only half of patients with asthma and in only 37% of patients with severe asthma [3, 4]. Non-T2 asthma is generally characterised by neutrophilic inflammation, also known as “steroid-resistant asthma” or severe asthma [5]. Accumulating evidence has suggested that Th17 is essential for the development of severe asthma [6–9]. Th17 asthma is generally characterised by a steroid-resistant, refractory asthma. Th17 cytokines are key players in T2-low disease, with increased levels of IL-17A and IL-17F found in the bronchial walls of severe asthmatics and associated with neutrophilic infiltration, AHR and steroid resistance [10, 11]. Moreover, neutrophils participate in the exacerbation of asthma through “NETosis”, which is a specific form of cell death in neutrophils [12]. Retinoic acid receptor-related orphan nuclear receptor gamma t (RORγt) is a transcription factor of Th17 cells, which mainly promotes the differentiation of Th17 cells [13]. Although previous studies have shown that pro-inflammatory cytokines, oxidative stress and epigenetic regulation are involved in Th17 cell-mediated severe asthma, its potential mechanism is still unclear.

MicroRNA(miRNA, miR) is a non-coding RNA with a length of 19–22 nucleotides. miRNA can inhibit gene translation by binding to the 3'-untranslated region (3'-UTR) of mRNA and then reduce the expression of target genes [14]. Accumulating evidence has shown that the critical roles of miRNAs in development and the underlying disease mechanism associated with their dysregulation have become increasingly known [15]. Numerous studies have shown that miRNAs are expressed in different relevant airway cell types to produce different asthma phenotypes involved in asthma pathogenesis, including airway inflammation, airway remodelling, the induction of steroid resistance and impaired innate antiviral immune response [16–22]. miR-146a-3p is expressed by leukocytes and its function is clearly associated with inflammation and innate immunity [23, 24]. Studies have shown a decrease in miR-146a expression in peripheral blood mononuclear cells (PBMCs) and the plasma of systemic lupus erythematosus (SLE) patients, suggesting that miR-146a has anti-inflammatory properties [25, 26]. It has been shown that miR-146a is upregulated in the skin and keratinocytes of atopic dermatitis (AD) patients, which alleviates chronic inflammation in a mouse model of AD [27–29]. Moreover, studies have demonstrated that miR-146a inhibits many pro-inflammatory chemokines in keratinocytes by targeting multiple factors of the NF-κB pathway [30, 31] and miR-146a-deficient mice develop stronger inflammation in both AD and psoriasis models [27, 32]. It has been shown that miR-146a-3p is upregulated in LPS-induced BEAS-2B cells and acute lung injury (ALI) rats and depleting miR-146a-3p improves ALI by up-regulating SIRT1 and mediating the NF-κB pathway [33]. MiR-146a and miR-146b are upregulated in the lung tissue of a mouse model of common allergic asthma [34]. Also, another study showed that miR-146a and miR-146b were upregulated in human airway smooth muscle from asthmatic subjects treated with cytomix (IL-1β, TNF-γ and IFNβ), suggesting that miR-146 mimics may be an attractive candidate for further preclinical studies as an anti-inflammatory treatment in asthma [35]. Circulating microRNAs (miR-146b, miR-206 and miR-720) are used in combination as potential prediction markers of asthma exacerbation in childhood asthma [36]. More critically, it has been shown that miR-146a is downregulated in bronchial brushing samples of asthma patients and also inhibits IL-8 and CXCL1 expression and neutrophil migration [37]. In spite of miR-146a-3p having been previously shown to suppress inflammatory responses in different diseases, tissues and cells, its potential pathomechanism in severe asthma is still unclear, especially the effect of Th17 cells differentiation.

The methyl-CpG-binding domain2 (MBD2) proteins are members of the methylated CpG binding protein family which can recognise the DNA methylation imprint and regulate their expression, thus making them critical mediators of many epigenetic processes [38, 39]. It has been shown that Th17 cells differentiation is insufficient in MBD2 knockout mice due to the unsuppressed T-bet/Hlx axis, resulting in protection from experimental autoimmune encephalomyelitis (EAE) [40]. Our previous work indicates that MBD2 mediates Th17 differentiation in a severe asthma mouse model through different cytokines [7, 9, 41], with further investigations revealing that MBD2 and Th17 are increased in the peripheral blood of severe asthma patients, while MBD2 was positively correlated with Th17, which indicates that MBD2 is a potential novel biomarker for identifying severe asthma with different endotypes [42].

In this study, we show that miR-146a-3p expression is downregulated in peripheral blood mononuclear cells of severe asthma patients. Based on the above reviews and miRNA databases, we hypothesise that miR-146a-3p would mediate Th17 differentiation in severe asthma by targeting MBD2. We have demonstrated that miR-146a-3p mediates Th17 differentiation by inhibiting MBD2, which binds to the MBD2 3’-UTR. Furthermore, we show that the inhalation of miR-146a-3p agomir suppresses the development of airway inflammation and relieves the severe asthma mouse model in vivo by decreasing MBD2 expression. Taken together, these data indicated that miR-146a-3p might be a potential novel therapeutic by targeting MBD2 in Th17 cell-mediated severe asthma.

## Materials and methods

### Subjects

All clinical specimens were obtained from severe asthma patients and healthy controls between April 10 2020 and February 20 2021 in The Second Xiangya Hospital of Central South University (Hunan, China). The diagnostic criteria for asthma were applied according to the Global Initiative for Asthma Guidelines (GINA); severe asthma requires treatment with guideline suggested medications for GINA steps 4–5 asthma (high-dose inhaled corticosteroids and LABA or leukotriene modifier/theophylline) for the previous year or systemic corticosteroids for > 50% of the previous year to prevent it from becoming “uncontrolled” or which remains “uncontrolled” despite this therapy [43], while the classification of mild, moderate and severe asthma was evaluated according to ACT, the pulmonary function test (PFT) and the therapeutic regimens of asthma patients [44, 45]. None of the subjects used statins or systemic corticosteroids for 3 months before entering the experimental group in the clinical study. The following patients were excluded: (a) acute episode, bronchogenic carcinoma, cardiac asthma, allergic bronchopulmonary aspergillosis; (b) atopic dermatitis, allergic rhinitis or other allergy-related conditions; (c) those suffering from autoimmune disorders, systemic lupus erythematosus (SLE), haematological diseases or serious infections; (d) those with disease complicated with malignancies or solid tumours; and (f) pregnant or lactating women. Healthy controls had no history of chronic respiratory disease and atopy, which is defined as an individual and/or familial tendency, usually in childhood or adolescence, to become sensitised and produce IgE antibodies in response to exposure to allergens, usually proteins [46]. Peripheral blood was collected from each patient within 2 h of admission to the hospital with a heparin vacuum tube and was used for the subsequent experiments (5 ml of blood per tube). None of the subjects accepted any drug or treatment before peripheral blood collection. According to the percentage of Th2 and Th17 cells in the pe-ripheral blood and by the type of inflammation, severe asthma was divided into T2 severe asthma and Th17 severe asthma. The percentage of Th2 and Th17 cells in the peripheral blood was measured by flow cytometry. The experiments on the use of human clinic samples were carried out after approval from the Ethics Committees of The Second Xiangya Hospital of Central South University (Hunan, China) as well as obtaining informed consent from all subjects.

### Isolation of peripheral blood mononuclear cells and flow cytometry

PBMCs were isolated from the peripheral blood of healthy human volunteers and severe asthma patients by density centrifugation with Ficoll-Paque [47, 48]. We used the human peripheral blood lymphocyte separation solution kit (TBD sciences, Tianjin, China), performed according to the manufacturer's instructions. Then, one tube of PBMCs mixed with Trizol (Invitrogen, USA) was stored at − 80 °C for qRT-PCR. Another tube of PBMCs was incubated for 6 h, before cells were stained with a marker of cell viability (Fixable Viability Stain 510 antibody) for 15 min at room temperature in the dark. Then, cells were stained for surface markers with APC-Cy7-anti-CD3 and BB515-anti-CD4 antibodies (BD Pharmingen) and fixed and permeabilised using the Cytofix/Cytoperm Soln Kit (BD Pharmingen) at 4 °C in the dark for 30 min. After washing with permeabilisation buffer, cells were stained for intracellular markers with PE-anti-IL-17A and APC-anti-IL-4 antibodies (BD Pharmingen) in permeabilisation buffer at 4 °C in the dark for 30 min. Isotype controls were employed in the control group. Flow cytometry was performed using the FACS Calibur (BioLend, USA). The data were analysed by FlowJo V10 software.

### Enzyme-linked immunosorbent assay (ELISA)

The expression of MBD2 in the peripheral blood serum of volunteers was measured using an ELISA kit (Genie, USA) according to the manufacturer's instructions.

### BECs isolation and culture

Murine BECs were isolated by cold enzymatic digestion of murine bronchi or tracheas as described previously [49, 50]. Single-cell suspensions from mice murine bronchi or tracheas were cultured in 12-well plates that were coated with collagen I (50 μg/ml, BD Biosciences, Franklin Lakes, New Jersey, USA) at 3.5 × 10^5^ cells/ml of MTEC (HyClone, USA) proliferation media containing RPMI-1640 medium (Gibco-ThermoFisher Scientific, USA), 10% heat-inactivated FBS, retinoic acid stock B (10 mmol/l, Sigma-Aldrich), insulin solution (6.25 mg/l, Sigma, USA), epidermal growth factor solution (50 ng/ml, BD Biosciences), bovine pituitary extract (25 mg/l; Sigma-Aldrich), transferrin solution (6.25 mg/l, Sigma-Aldrich) and cholera toxin solution (4.2 mg/l, Sigma-Aldrich). The submerged MTEC cultures were incubated at 37 °C in a humidified incubator containing 95% air and 5% CO_2_. After 6 h, the supernatant and non-adherent cells were discarded. The adherent cells were allowed to differentiate for 7–10 days by replacing the proliferation medium with MTEC basal medium containing Nu-seum (2%, BD Biosciences) and retinoic acid (10 mmol/l, Sigma–Aldrich). BECs were centrifuged and stained with cytokeratin-specific monoclonal antibody and DAPI.

### HDM/LPS exposure model of BECs and transfection

The Th17 predominant cellular severe asthma model was induced as previously reported, where BECs were irritated with 100 μg/ml of house dust mite (HDM) (Indoor Biotechnologies, USA) + 100 ng/ml of lipopolysaccharide (LPS) (Solarbio Life Sciences, Beijing, Beijing, China), or PBS for 24 h [51]. Mimic miR-146a-3p, negative control (mimic NC), inhibitor miR-146a-3p, negative control (inhibitor NC), small interfering RNA targeting MBD2 (si-MBD2) and negative control (si-NC) were synthesised by RiboBio Co., Ltd. (Guangzhou, China). The mmu-miR-146a-3p primer sequences were 5′-CGCGCCTGTGAAATTCAGTTCTTCAG-3′ (forward). The MBD2 interfering sequence was 5′-GCAAGATGATGCCTAGTAA-3′. MBD2 overexpression plasmid (OE-MBD2) and negative control (OE-NC) were purchased from HonorGene (Changsha, China). They were transfected separately into BECs with Lipofectamine™ 3000 (Thermo Fisher Scientific), for 24 h at 37 °C in a humidified incubator containing 95% air and 5% CO_2_. Finally, 48 h later, cells were treated with 100 µg/ml HDM and 100 ng/ml LPS for 24 h.

### Naive CD4 + T cell isolation and cocultivation

Spleen naive CD4 + T cells of nomal mice were isolated by magnetic bead cell sorting (MACS) (Miltenyi Biotec, Germany) using a CD4+ naive T-cell isolation kit (Stemcell Technologies, Vancouver, British Columbia, Canada) according to the manufacturer’s guidelines. BECs were irritated with 100 μg/ml of HDM + 100 ng/ml of LPS, or PBS for 24 h, after which BECs and CD4 + T cells were co-cultivated at a ratio of 10:1 (TCs: BECs) in RPMI-1640 medium supplemented with 10% FBS and containing soluble anti-CD3e (0.5 μg/ml; eBioscience, Waltham, Massachusetts, USA), soluble anti-CD28 (1.0 μg/ml; eBioscience) and IL-2 (20 ng/ml; eBioscience). After 24 h, the suspended cells were collected to analyse CD4, IL-4 and IL-17A concentrations determined by flow cytometry.

### Flow cytometry

After co-culture, navie CD4 + T cells were incubated with leukocyte activation cocktail (100 ng/ml of PMA, 1 μg/ml of ionomycin and 2 μM monensin) for 6 h (BD Biosciences, Franklin Lakes, New Jersey, USA). The cells were then fixed and permeabilised with fixation and permeabilisation buffer (Multi Sciences Company) for 15 min. After washing with permeabilisation buffer, the cells were stained with the intracellular markers APC-anti-IL-17 and PE-anti-IL-4 cytokine antibodies (BioLend, USA) in permeabilisation buffer for 20 min. Flow cytometry was conducted using the FACS Calibur (BioLend, USA). The data were analysed by FlowJo V10 software.

### Luciferase reporter assay

The wild-type (WT) MBD2 3′-untranslated region (3′-UTR) was amplified by PCR and cloned into the psiCHECK-2 vector (Promega, Madison, USA). MBD2 cloned to the psiCHECK-2 vector was mutated to obtain the mutant type (MT) MBD2 using the Easy Mutagenesis System Kit (Promega, Madison, USA). Then, 293-T cells were transfected with the WT-MBD2-3′-UTR or MT MBD2-3′-UTR plasmid (300 ng) and mimic miR-NC or mimic miR-146a-3p (100 nM), using Lipofectamine™ 3000 (Thermo Fisher Scientific) transfection reagent based on the manufacturer’s instructions. Forty-eight hours after transfection, a luciferase assay was performed using a dual-luciferase reporter assay kit according to the manufacturer’s instructions (Keygen biotech, Changsha, China).

### Quantitative real-time polymerase chain reaction (qRT-PCR)

Total RNA was isolated from harvested cells and tissues with TRIzol (Invitrogen, USA) following the manufacturer’s protocols. Reverse-transcription quantitative polymerase chain reaction (RT-qPCR) was performed using the miRNA First Strand cDNA Synthesis (Tailing Reaction) kit (Sangon Biotech, Shanghai, China) to examine the relative miR-146a-3p expression, in accordance with the manufacturer’s instructions. U6 was used for normalisation. Quantitative real-time PCR was performed using the Fast sTaq PCR Master Mix (servicebio, Wuhan, China). The comparative threshold cycle (Ct) value method was used to analyse relative gene expression.

### Western blotting

Total proteins were prepared using RIPA lysis buffer supplemented with protease inhibitors. Western blotting was carried out by probing the membranes with indicated primary antibodies followed by incubating with an HRP-conjugated secondary antibody.

### Mice model of Th17 predominant neutrophilic severe asthma and miR-146a-3p aerosol inhalation

All studies were performed in compliance with the Second Xiangya Hospital, Central South University Animal Care and Use Committee. Wild-type C57BL/6 mice were purchased from HUNAN SJA Laboratory Animal Co. LTD (Changsha, China). Female mice, aged 6–7 weeks, weighing about 20 g, were used in the experiments. All mice were bred and housed in an SPF facility with a 12/12 h light/dark cycle. The group of severe asthma model mice were given an intraperitoneal sensitisation injection with 100 µg house dust mites (HDM, Greer Laboratories, USA), 100 µg Ovalbumin (OVA, Grade V, Sigma-Aldrich, USA) and 15 µg lipopolysaccharide (LPS, Sigma) with 2 mg aluminium hydroxide (Sigma) on days 0, 1 and 2; mice were then rechallenged with OVA solution atomised for 30 min before HDM intranasal excitation on days 14, 15, 18 and 19, as previously reported [52]. For the saline group, mice received the same procedures as severe asthma, but were given saline instead of drugs. The severe asthma model mice were then randomly divided into the following 3 groups (8 mice each): severe asthma + saline, severe asthma + negative control of agomir and severe asthma + miR-146a-3p agomir groups. The miR-146a-3p agomir dosage was 10 nmol for each mouse and sprayed into the lung at 13 days and then before every rechallenge (24 h before each HDM rechallenge) until mice were sacrificed at day 21 using a Penn-Century Micro-Sprayer (Penn-Century Inc.) [19, 53].

### Airway hyperresponsiveness

On day 21 (24 h after the final challenge), baseline (RL0) and methacholine (Mch) induced airway hyperresponsiveness measured by Buxco Electronics, RC System, USA plethysmograph, as previously described [54] in tracheotomised intubated mice after anaesthesia. Then, 10 μl Mch was mixed with 10 μl saline for the initiation of broncho-provocation and Mch was increased to 0.39 mg/ml (dose 1), 0.78 mg/ml (dose 2), 1.56 mg/ml (dose 3) and 3.12 mg/ml (dose 4), with the change in lung resistance (RLX) recorded for analysis.

### BALF (Broncho-Alveolar lavage fluids) cell count

BALF was collected as described previously [54]. A haemocytometer determined total BALF cell counts after the removal of red blood cells by centrifuge and precipitation. After cell  smear and H&E staining, BALF NEU (neutrophils) and EOS (eosinophils) were determined in 200 total BALF cells.

### Histopathology

Lung tissues were fixed in 10% neutralised buffered formalin (Nacalai Tesque) and embedded in paraffin. Sections were subjected to H&E or periodic acid-Schiff staining. For the immunohistochemical staining of lung tissues, rehydrated antigen-retrieved sections were incubated with anti-MBD2 antibody, anti-ECP antibody and anti-LY6G antibody and visualised using the avidin–biotin complex method with the chromogen diaminobenzidine (Vector Laboratories). Quantification was performed using automated computerised image analysis (CellSens dimension: Olympus).

### Statistics

SPSS 26.0 software (IBM Corp.) was used to perform all statistical analyses. Continuous variables were described as the mean and standard deviation (M ± SD) or median (interquartile range [IQR]). Statistical analysis was performed by Student's t-test to determine the significance of the differences between the control and experimental groups, one-way analysis of variance (ANOVA) or Kruskal–Wallis test followed by Dunn’s multiple comparisons test. Differences were considered statistically significant when p < 0.05.

## Results

### Subject characteristics

As shown in Table 1, we recruited 30 healthy control volunteers (HCs) and 30 severe asthma patients (SA). All volunteers were of Chinese origin, strictly following the inclusion and exclusion criteria. There were no significant differences in age (HCs 56.90 ± 11.57, SA 60.43 ± 9.03), sex (HCs 20/10, SA 24/6) or BMI (HCs 23.69 ± 2.97, SA 22.11 ± 3.53) between the two groups. Asthma control was assessed with an asthma control test (ACT 12.80 ± 3.17), which indicated that the patient felt symptomatic and that their asthma was poorly controlled. Severe asthma mainly includes T2 and Th17 asthma. Peripheral blood mononuclear cells were examined by flow cytometry to distinguish between T2 and Th17 severe asthma (Supplementary Figure). Table 2 shows the demographic characteristics, pulmonary function indexes and biochemical indexes of the 20 T2 severe asthma patients and 10 Th17 severe asthma patients. There were no significant differences in age, pulmonary function or ACT between T2 severe asthma patients and Th17 severe asthma patients. However, compared to T2 severe asthma patients, Th17 severe asthma patients had a higher percentage of Th17 cells with a lower percentage of Th2 cells, meaning more neutrophils but fewer eosinophils. Interestingly, Th17 severe asthma appears to predominate in obese women.

**Table 1:** Clinical characteristics of participants

Items	Control (n = 30)	Severe asthma (n = 30)	p value
Age(y), M ± SD	56.90 ± 11.57	60.43 ± 9.03	0.19
Sex M/F	20/10	24/6	0.24
FEV1(L)	2.94 ± 0.88	1.02 ± 0.32	0.00
FEV1%pred, M ± SD	100.74 ± 11.60	38.26 ± 9.65	0.00
FEV1/FVC, M ± SD	80.20 ± 3.51	41.32 ± 8.19	0.00
BMI (kg/m^2^), M ± SD	23.69 ± 2.97	22.11 ± 3.53	0.07
ACT, M ± SD		12.80 ± 3.17	
Eosinophils (× 109)	0.14 ± 0.11	0.31 ± 0.28	0.02
Neutrophils (× 109)	3.50 ± 0.80	4.73 ± 1.74	0.01

Chi-square test, one-way analysis of variance (ANOVA) or Kruskal–Wallis test for comparisons followed by Dunn’s multiple comparisons test. p < 0:05 was considered statistically significant

FEV1, forced expiratory volume in 1 s; FEV1%pred, forced expiratory volume in 1 s% prediction; FVC, forced vital capacity; M ± SD, mean ± standard deviation; M/F, men/ female; BMI, body mass index; ACT, asthma control test

**Table 2:** Clinical characteristics of T2 and Th17 severe asthma participants

Items	T2 severe asthma (n = 20)	Th17 Severe asthma (n = 10)	p value
Age (y), M ± SD	60.70 ± 8.05	59.9 ± 11.02	0.824
Sex M/F	19/1	5/5	0.04
FEV1(L)	0.99 ± 0.35	1.06 ± 0.25	0.57
FEV1%pred, M ± SD	36.89 ± 9.9	41.00 ± 8.96	0.28
FEV1/FVC, M ± SD	41.25 ± 9.04	41.47 ± 6.59	0.95
BMI (kg/m^2^), M ± SD	22.85 ± 3.85	26.61 ± 3.23	0.013
ACT, M ± SD	13.15 ± 3.54	12.81 ± 2.23	0.40
Eosinophils (× 109)	0.44 ± 0.26	0.23 ± 0.16	0.020
Neutrophils (× 109)	4.43 ± 1.62	6.24 ± 1.28	0.005
Th2 (%), M ± SD	3.84 ± 0.35	3.24 ± 0.39	0.00
Th17 (%), M ± SD	2.95 ± 0.44	4.00 ± 0.48	0.00

Chi-square test, one-way analysis of variance (ANOVA) or Kruskal–Wallis test for comparisons followed by Dunn’s multiple comparisons test. p < 0:05 was considered statistically significant. Abbreviations: FEV1, forced expiratory volume in 1 s; FEV1%pred, forced expiratory volume in 1 s% prediction; FVC, forced vital capacity; M ± SD, mean ± standard deviation; M/F, men/ female; BMI, body mass index; ACT, asthma control test

### Differential expression of miR-146a-3p in PBMCs and MBD2 in peripheral blood serum of severe asthma patients

**Fig. 1:**
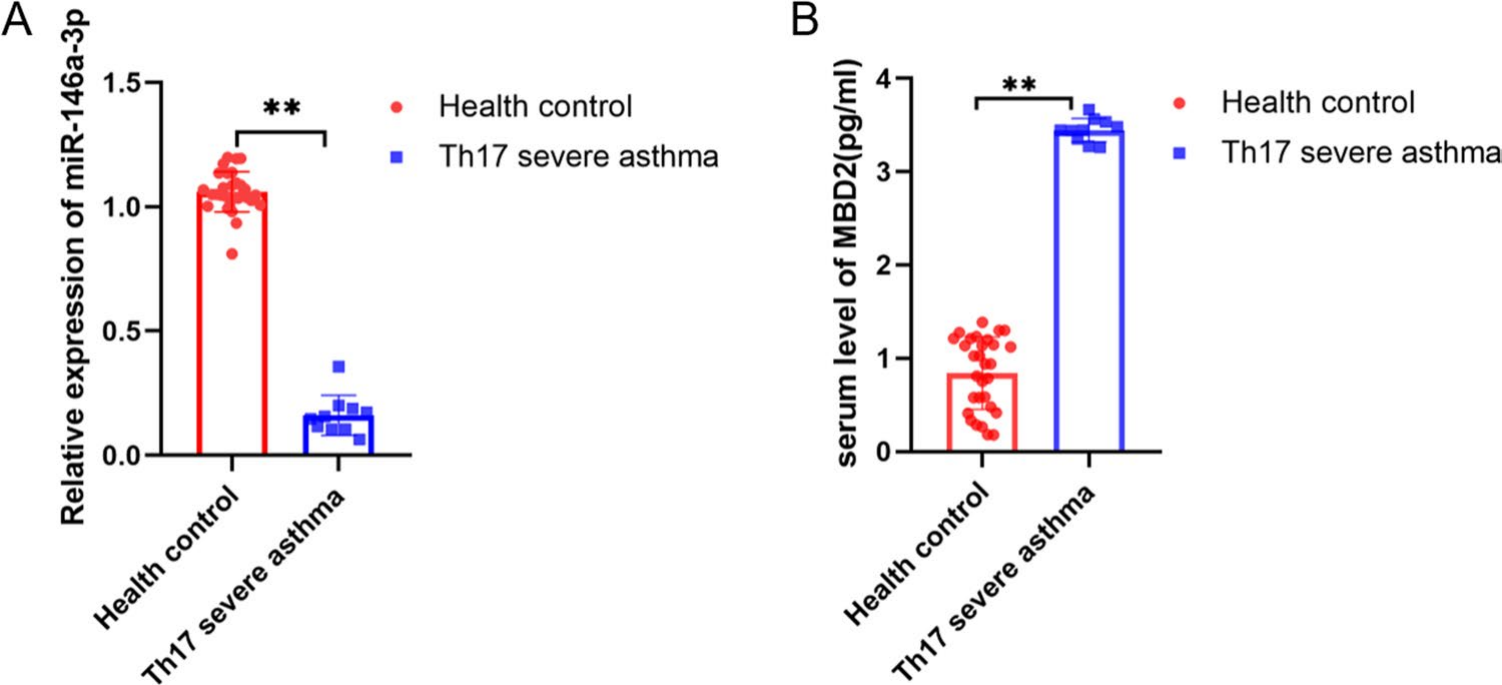
The differential expression of miR-146a-3p and MBD2 in Peripheral blood serum. **A** the relative expression of miR-146a-3p in PBMCs of  Th17 severe asthma patients by Q-PCR. **B** the expression of MBD2 in serum of Th17 severe asthma patients by ELISA (**p < 0.01)

The relative expression of miR-146a-3p in PBMCs of  Th17 severe asthma was significantly downregulated compared to healthy controls (p < 0.01) (Fig. 1A). What is more, we measured the expression of MBD2 in peripheral blood serum by ELISA; the results showed that the expression of MBD2 was clearly increased in severe asthma patients compared to healthy controls (p < 0.01) (Fig. 1B).

### The differential expression of miR-146a-3p in  HDM/LPS exposure model of BECs

**Fig. 2:**
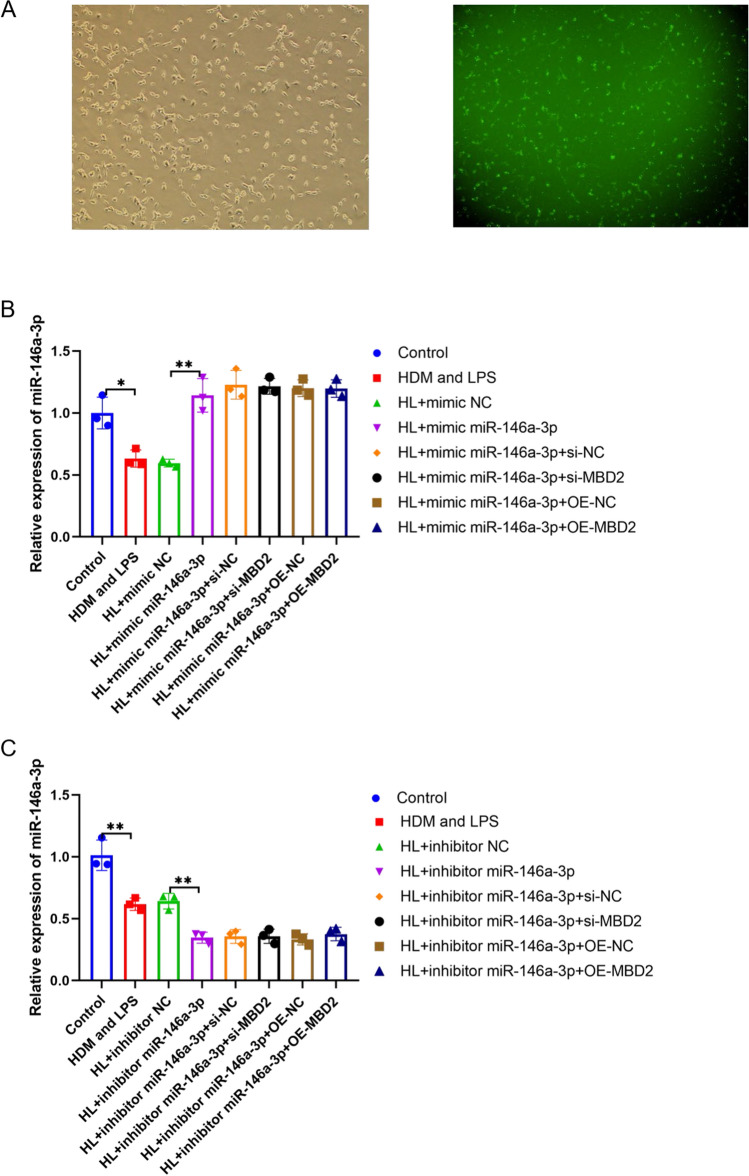
The differential expression of miR-146a-3p in BECs. **A** the transfection rate of mimic miR-146a-3p labelled with green fluorescence in BECs by immunofluorescence. **B** the relative expression of miR-146a-3p in BECs after irritated with HDM and LPS(HL), and transfected with mimic miR-146a-3p, negative control (mimic NC), small interfering RNA targeting MBD2 (si-MBD2), negative control (si-NC), MBD2 overexpression plasmid (OE-MBD2) and negative control (OE-NC) by Q-PCR. C, the relative expression of miR-146a-3p in BECs after irritated with HDM and LPS, and transfected with inhibitor miR-146a-3p, negative control (inhibitor NC), small interfering RNA targeting MBD2 (si-MBD2), negative control (si-NC), MBD2 overexpression plasmid (OE-MBD2) and negative control (OE-NC) by Q-PCR. U6 as an internal control (N = 3, * p < 0.05, ** p < 0.01)

We, respectively, overexpressed and inhibited miR-146a-3p and MBD2 in BECs and used immunofluorescence to detect the transfection rate. To evaluate transfection efficiency, the mimic miR-146a-3p was labelled with green fluorescence, immunofluorescence identification results showed that the transfection rate of the mimic miR-146a-3p was more than  90% (Fig. 2A). The relative expression of miR-146a-3p was downregulated in BECs irritated with HDM and LPS (p < 0.05). After transfection with the mimic miR-146a-3p, the relative expression level of miR-146a-3p was significantly increased (p < 0.01); however, when mimics miR-146a-3p and si-MBD2 or OE-MBD2 were simultaneously transfected, the expression of miR-146a-3p did not change (Fig. 2B). After being transfected with the inhibitor miR-146a-3p, the relative expression level of miR-146a-3p was decreased (p < 0.01); however, when the inhibitors miR-146a-3p and si-MBD2 or OE-MBD2 were simultaneously transfected, the expression of miR-146a-3p did not change (Fig. 2C).

### miR-146a-3p directly targets MBD2 3′-UTR

**Fig. 3:**
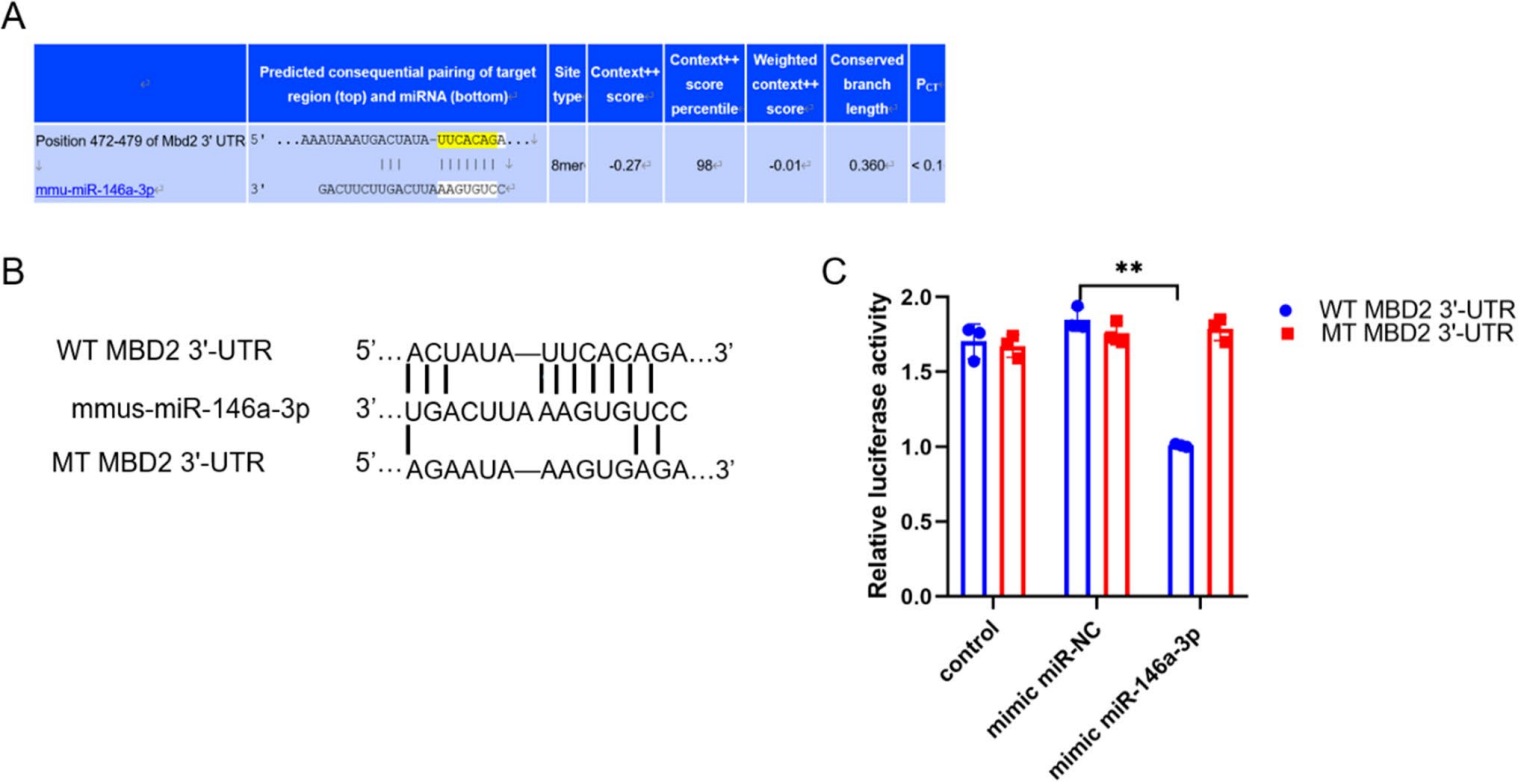
MiR-146a-3p directly targets MBD2 3’-UTR. **A** MBD2 was predicted to be a potential target gene of mmu-miR-146a-3p with evolutionary conservation. **B** The wild-type MBD2 3′-UTR containing the putative miR-146a-3p binding sites and mut-type MBD2 3′-UTR without. **C** mimic miR-146a-3p effectively inhibited WT-MBD2 3′-UTR luciferase reporter activity compared to the mimic miR-146a-3p NC, whereas mut-type MBD2 3′-UTR abolished this effect by dual-luciferase reporter assay (**p < 0.01)

We performed a bioinformatic analysis using miRanda and TargetScan, which showed that MBD2 may be a target gene of miR-146a-3p (Fig. 3A). To demonstrate that MBD2 is a target gene of miR-146a-3p, the wild-type and mut-type MBD2 3′-UTR were subcloned into the psiCHECK-2 MBD2 luciferase reporter vector (Fig. 3B). Then, mimic miR-146a-3p and psiCHECK-2 MBD2 luciferase reporter vectors were simultaneously transfected into 293 T cells and the luciferase reporter assay was performed. The results show that mimic miR-146a-3p effectively decreased WT-MBD2 3′-UTR luciferase reporter activity compared to the mimic miR-NC. Nonetheless, after being co-transfected with the MT MBD2 3′-UTR vector and mimic miR-146a-3p, the luciferase activity was not altered in 293 T cells (Fig. 3C). These results confirmed that MBD2 was a direct target of miR-146a-3p.

### miR-146a-3p mediates Th17 differentiation by targeting the MBD2 in HDM/LPS exposure model of BECs

**Fig. 4:**
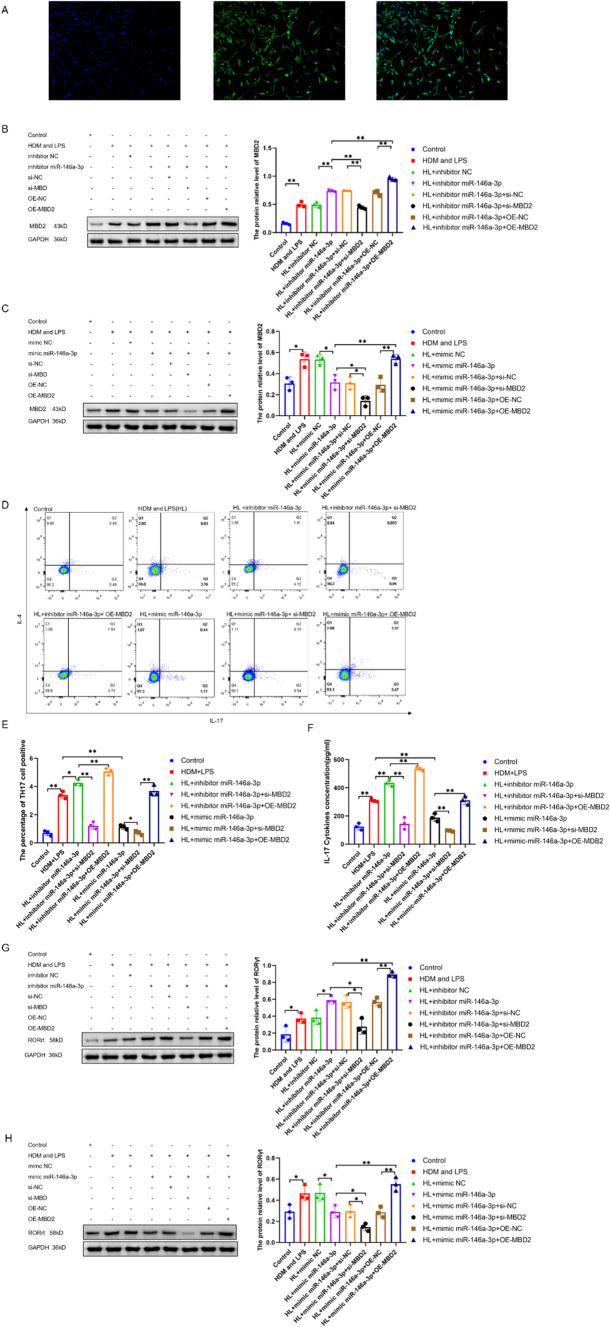
miR-146a-3p mediates Th17 differentiation by targeting the MBD2 in BECs. **A** the positive rate of primary culture of mouse BECs by immunofluorescence. **B** the protein expression of MBD2 in BECs after irritated with HL, and transfected with inhibitor miR-146a-3p, inhibitor NC, si-MBD2, si-NC, OE-MBD2 and OE-NC by western blotting. **C** the protein expression of MBD2 in BECs after irritated with HL, and transfected with mimic miR-146a-3p, mimic NC, si-MBD2, si-NC, OE-MBD2 and OE-NC by western blotting. **D** and **E** the percentage of Th17 cell positive in CD4 + T cell cocultivation with BECs after irritated with HL and transfected with inhibitor miR-146a-3p, mimic miR-146a-3p, si-MBD2, OE-MBD2 by flow cytometry. **F** the IL17 cytokines concentration in cell culture fluid of different groups by ELISA. **G** the protein expression of RORγt in CD4 + T cell after irritated with HDM and LPS, and transfected with after irritated with HL, and transfected with inhibitor miR-146a-3p, inhibitor NC, si-MBD2, si-NC, OE-MBD2 and OE-NC by western blotting. G, the protein expression of RORγt in after irritated with HL, and transfected with mimic miR-146a-3p, mimic NC, si-MBD2, si-NC, OE-MBD2 and OE-NC by western blotting. GAPDH as an internal control (N = 3, * p < 0.05, ** p < 0.01)

To further explore miR-146a-3p-mediated Th17 differentiation in severe asthma by targeting MBD2, we isolated murine BECs from murine bronchi or tracheas. After isolation, immunofluorescence identification results showed that the rate of cytokeratin positive cell population of BECs in mice was more than 90% (Fig. 4A).  Then BECs were then irritated with HDM and LPS and transfected with the inhibitor miR-146a-3p, mimic miR-146a-3p, si-MBD2, OE-MBD2. After that, cells were co-cultivated with CD4 + T cells to examine Th17 differentiation by flow cytometry. The Western blotting data showed that the protein relative of MBD2 increased after being irritated with HDM and LPS(HL) and further increased after simultaneous transfection with the inhibitor miR-146a-3p but decreased after simultaneous transfection with the inhibitors miR-146a-3p and si-MBD2 (p < 0.01). However, after simultaneous transfection with the inhibitors miR-146a-3p and OE-MBD2, the relative protein levels of MBD2 were significantly increased (Fig. 4B). Meanwhile, after being irritated with HL and transfected with the mimic miR-146a-3p, the expression of MBD2 was significantly decreased, but further decreased after being simultaneously transfected with mimics miR-146a-3p and si-MBD2 (p < 0.05). After simultaneous transfection with mimic miR-146a-3p and OE-MBD2, the relative protein level of MBD2 increased (Fig. 4C). The flow cytometry data showed that the percentage of Th17-positive cells increased after being irritated with HL and further increased after simultaneous transfection with the inhibitor miR-146a-3p (p < 0.05), but was decreased after being simultaneously transfected with si-MBD2 and increased after simultaneous transfection with OE-MBD2 (p < 0.01). Meanwhile, after irritation with HL and transfected mimic miR-146a-3p, the percentage of Th17-positive cells significantly decreased and further decreased after simultaneous transfection with si-MBD2 (p < 0.05) and increased after being simultaneously transfected with OE-MBD2 (p < 0.01) (Fig. 4D,E). In addition, the IL17 cytokine concentration in cell culture fluid detected by ELISA also showed the same result as the percentage of Th17-positive cells in the different groups (Fig. 4F). Furthermore, RORγt, the master transcription factor for Th17 differentiation, was analysed. The protein relative of RORγt in CD4 + T cell showed the same result as the percentage of Th17-positive cells in the different groups (Fig. 4G,H). Taken together, these data indicated that miR-146a-3p mediates Th17 differentiation by targeting the MBD2 in Th17 predominant neutrophilic severe asthma.

### miR-146a-3p as a potential therapeutic target for Th17 predominant neutrophilic severe asthma in vivo

**Fig. 5:**
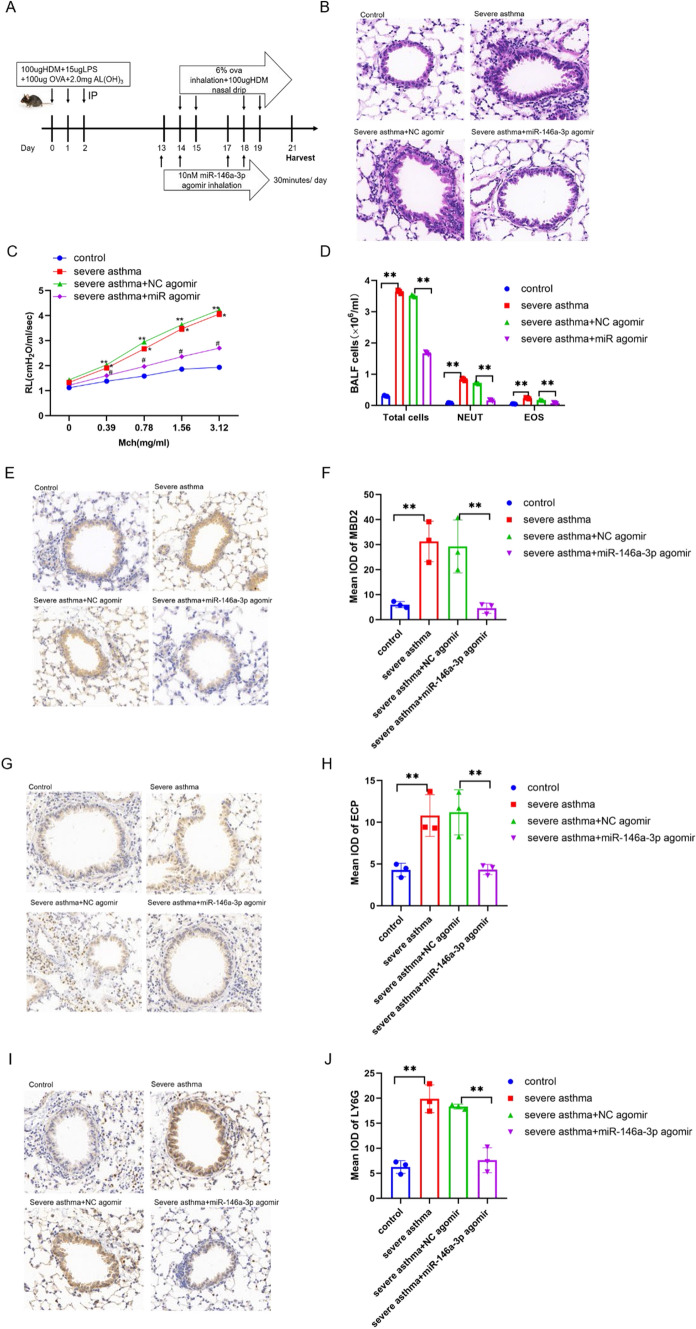
MiR-146a-3p as a potential therapeutic target for severe asthma in vivo. **A** Scheme of the experimental asthma model. Wild-type C57BL/6 mice were sensitised with HDM/LPS/OVA/AL(OH)3 or PBS on days 0,1,2. The mice were nebulized with OVA and dripped nasal with HDM on days 14,15,18,19, meanwhile nebulized with miR-146a-3p agomir before 24 h before each rechallenges, and analysed on day 21. **B** Lung tissues stained with H&E, Scale bar, 50 μm. C, Airway hyperresponsiveness represented by the increased value of pulmonary resistance (**p < 0.01 versus control, *p < 0.01 versus control, #p < 0.01 versus NC agomir). **D** total, neutrophil and eosinophil cells in BALF in the different groups of mice. **E**–**G** IHC results of MBD2(E), ECP(G) and LY6G(I) staining, quantification of MBD2(F), ECP(H) and LY6G (J)staining integrated optical density (IOD) in lung tissues, Scale bar, 50 μm (**p < 0.01)

To explore whether miR-146a-3p can act as a potential therapeutic in vivo, we established a mouse model of severe asthma and synthesised miR-146a-3p agomir, which was inhaled into the lungs of mice 24 h before each HDM rechallenge (Fig. 5A). Eosinophils and neutrophils infiltrated around the bronchi in severe asthma, with neutrophils predominating. However, after treatment with miR-146a-3p agomir, a decrease was observed in mucus secretion, inflammatory cells and airway hyperresponsiveness (Fig. 5B). Airway resistance in mice from each group was detected using the lung function detection system for small animals with increasing methacholine concentrations (from 0.39 mg/ml to 3.12 mg/ml). Airway resistance data showed that airway resistance increased in severe asthma mice compared to the control group. Nevertheless, after being treated with miR-146a-3p agomir, airway resistance decreased (Fig. 5C). The bronchoalveolar lavage fluid (BALF) results showed that total cells, eosinophils and neutrophils were all upregulated in severe asthma, neutrophils more significantly, compared to the control group, but when treated with miR-146a-3p agomir, these data decreased (Fig. 5D). We performed immunohistochemistry (IHC) on mouse lung cells and found increased MBD2 immunopositivity in severe asthma compared to control group, but this was dramatically decreased in the miR-146a-3p agomir group (Fig. 5E,F). Ly6g (neutrophil-specific antibody) (Fig. 5G,H), ECP (eosinophil cationic protein) (Fg. 5I,J) immunohistochemistry and BALF showed the same results, which indicated that neutrophil infiltration was dominant in severe asthma and miR-146a-3p could decrease inflammatory cell levels.

### miR-146a-3p inhibit Th17 differentiation treatment of severe asthma in vivo

**Fig. 6:**
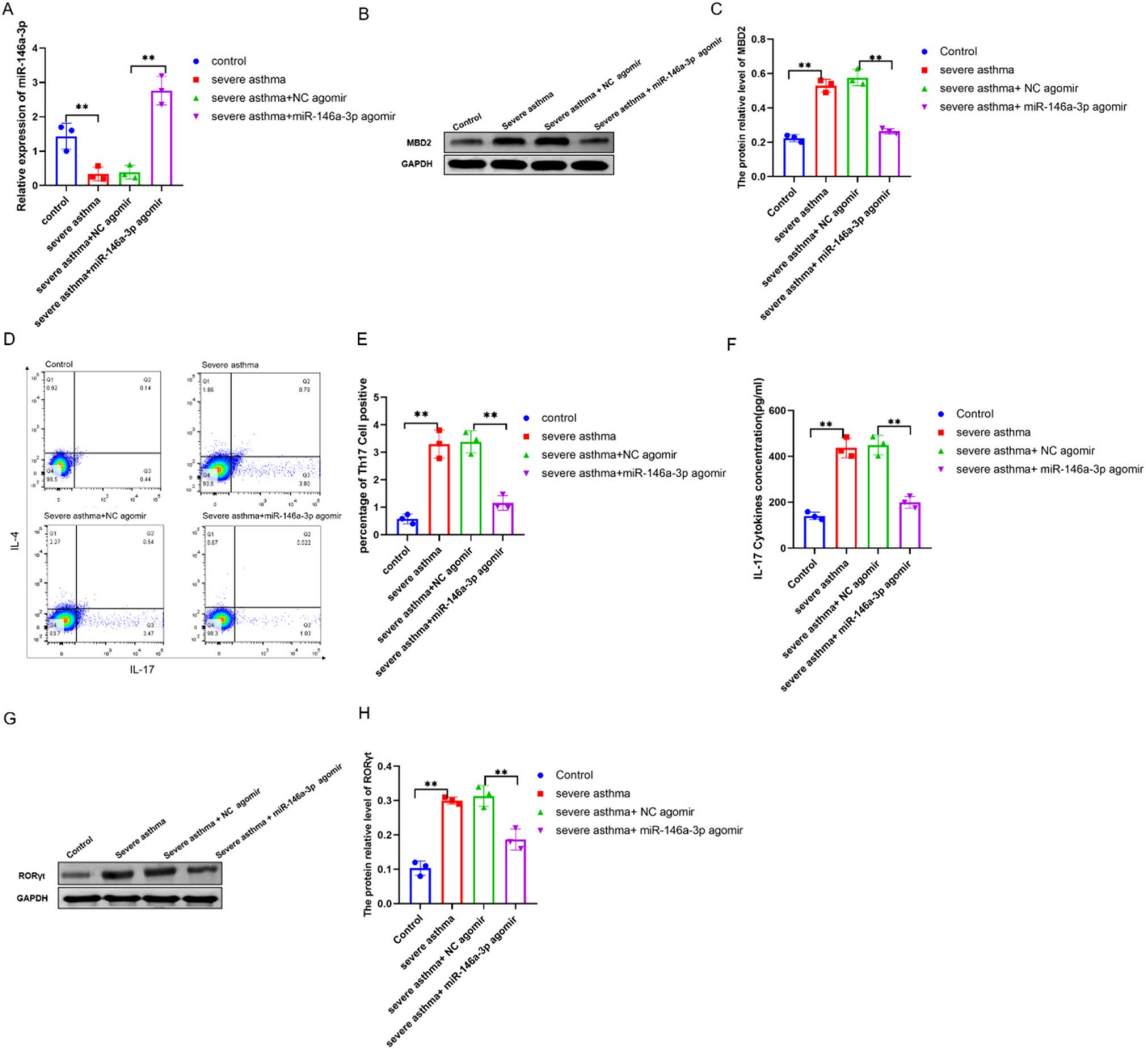
The expression of miR-146a-3p, MBD2 and Th17 differentiation in different group mice. **A** The relative expression of miR-146a-3p; **B** and **C** the protein relative expression of MBD2. **D** and **E** ratio of positive Th17 cells from spleen CD4 + T cells assessed by flow cytometry. **F** The IL17 cytokines concentration in BALF by ELISA. **G** and **H**. The protein relative expression of RORγt in spleen CD4 + T cells. (N = 3, **p < 0.01, *p < 0.05.)

We examined the relative miR-146a-3p lever in lung mouse, with results showing that the relative miR-146a-3p level was decreased in severe asthma, but significantly increased in the miR-146a-3p agomir group (Fig. 6A). Meanwhile, the relative protein level of MBD2 in mouse lungs increased in the severe asthma group compared to the control group and was decreased in the miR-146a-3p agomir group (Fig. 6B and 6C). To further explore the effect of miR-146a-3p on Th17 differentiation in vivo, we isolated spleen CD4 + T cells in the different groups of mice and examined Th17 differentiation by flow cytometry. We found that the percentage of Th17-positive cells was increased in severe asthma compared to the control group but decreased after treatment with miR-146a-3p (Fig. 6D and 6E). In addition, the IL17 cytokine concentration in BALF detected by ELISA also showed the same result as the percentage of Th17 -positive cells in the different groups (Fig. 6F). Furthermore, the relative protein level of ROR-γT in spleen CD4^+^ T cells increased in severe asthma but decreased after miR-146a-3p agomir was inhaled (Fig. 6G and 6H). These data suggested that miR-146a-3p inhibited Th17 differentiation in severe asthma by targeting MBD2.

## Discussion

In this study, we first demonstrated that miR-146a-3p decreased in PBMCs and MBD2 increased in the serum of subjects with severe asthma patients. Moreover, miR-146a-3p inhibited MBD2 expression by directly targeting the 3’-UTR. We found that miR-146a-3p suppressed Th17 differentiation by targeting MBD2 in BECs of the severe asthma model. While BECs had the function of antigen-presenting cells in vitro, our research for the first time reported that global miR-146a-3p significantly ameliorated severe asthma. miR-146a-3p overexpression significantly reduced airway hyperresponsiveness, airway inflammation, airway mucus secretion and inhibited Th17 cell response in vivo, which relieved severe asthma, providing a potential novel therapeutic in Th17 predominant severe asthma.

Glucocorticoid is an essential strategy in the treatment of asthma, according to GINA. However, hormone-sensitive asthma (Th2) is observed in only half of patients with asthma and only 37% of patients with severe asthma; also, Th17, which is seen in non-Th2 asthma, is essential for the development of hormone-resistant asthma or severe asthma. Therefore, there is an urgent need to develop new treatment strategies for severe or hormone-resistant asthma. Although our previous study found that MBD2 plays an important role in the pathogenesis of severe asthma and MBD2-KO significantly ameliorated severe asthma, MBD2 upstream epigenetic regulation in severe asthma remains unknown.

In this study, we found that MBD2 may be a target gene of miR-146a-3p by bioinformatic analysis [55]. A large number of studies have shown that miR-146a-3p has anti-inflammatory effects, while asthma can cause systemic inflammatory reactions. For that reason, we isolated mononuclear cells from peripheral blood in severe asthma and then detected the differential expression of miR-146a-3p in PBMCs by qRT-PCR. Interestingly, we found that the relative miR-146a-3p levels were markedly decreased in PBMCs, while MBD2 protein expression levels were increased in the serum of severe asthma patients, which is consistent with our previous research [42]. Our finding about miR-146a-3p is consistent with that of Kivihall et al., indicating that decreased levels of miR-146a in HBECs from patients with asthma may contribute to the development of the neutrophilic phenotype of asthma [37]; however, other studies have found that miR-146a was upregulated in the lung tissue of a mouse model of common allergic asthma [34]. It may be that different asthma phenotypes (common allergic asthma) lead to different results.

In order to verify the effect of miR-146a-3p on MBD2, we, respectively, overexpressed or inhibited miR-146a-3p and MBD2 in vitro and performed a Luciferase reporter assay. The results demonstrated that miR-146a-3p inhibited MBD2 expression by directly targeting MBD2 3’-UTR. miR-146a-3p may contribute to the development of the neutrophilic phenotype of asthma and Th17 is essential for the development of neutrophilic asthma. While BECs can serve as a kind of antigen-presenting cell to regulate the differentiation of T cells, we isolated murine BECs form murine bronchi or tracheas irritated with HDM and LPS and then examined relative miR-146a-3p levels in BECs before transfecting with the inhibitor miR-146a-3p or mimic miR-146a-3p. After that, cocultivation with CD4 + T cells to examine Th17 differentiation by flow cytometry was performed as described previously [51]. The MBD2 protein expression and percentage of Th17-positive cells increased in BECs irritated with HDM and LPS, which indicated that BECs can serve as a kind of antigen-presenting cell to regulate the differentiation of T cells. Although our results indicated that miR-146a-3p inhibited Th17 differentiation by targeting MBD2, it is not clear how MBD2 affects Th17 differentiation. Our previous research demonstrated that as an important role in methylation, MBD2 influenced Th17 differentiation through a variety of pathways [7, 9, 41]. Taking all together, we come to the conclusion that miR-146a-3p suppressed Th17 differentiation in severe asthma by targeting MBD2.

To further explore the effect of miR-146a-3p on Th17 differentiation in vivo, we constructed a Th17 predominant neutrophilic severe asthma mouse model and intervened with miR-146a-3p agomir inhalation 24 h before each HDM rechallenge. Our findings indicated that we successfully constructed a mouse severe asthma model and that after miR-146a-3p agomir inhalation intervention, bronchial inflammatory infiltration in the lung tissues of mice decreased, while asthma symptoms relieved and the positive rate of Th17 decreased. miR-146a negatively regulates severe inflammation during the innate immune response [56].

In this paper, we first demonstrated that relative miR-146a-3p levels decreased in the serum of subjects with severe asthma patients and miR-146a-3p inhibited Th17 cells differentiation, consistent with previous studies [37]. MiRNA is not only a regulatory factor in the pathogenesis of asthma, but also a therapeutic target for asthma. The selective blockade of miR-126 suppressed the asthmatic phenotype, resulting in diminished Th2 responses, inflammation, airway hyperresponsiveness, eosinophil recruitment and mucus hypersecretion [19]. The intranasal infusion of miR-155 antagonists significantly reduced the level of miR-155 in lung tissue and affects the function of lymphocytes in a mouse asthma model [57]. The knockout of miR-145 reduced airway eosinophil inflammation, airway mucus secretion, the production of Th2 cytokines and airway hyperresponsiveness [58]. The knockdown of miR-106a increased IL-10 production in lung tissues, significantly reduced airway hyperresponsiveness and airway inflammation and inhibited Th2 cell response, goblet cell metaplasia and airway subepithelial fibrosis [59]. The knockdown of miR-126 expression inhibited allergic airway inflammation induced by HDM, including a reduction of Th2 response, airway hyperresponsiveness, eosinophil aggregation and airway mucus secretion [60]. Kumar et al. injected exogenous let-7 mimic into the lungs of allergic inflammatory mice, which resulted in the decrease in IL-13 levels, airway inflammation, airway hyperresponsiveness, goblet cell metaplasia and subepithelial fibrosis [61]. miR-146a is an endogenous regulatory factor with anti-inflammatory effects and the application of miR-146a mimics can significantly reduce the inflammatory level of airway smooth muscle cells and Th2 lymphocytes in asthma [35].

To date, studies of miRNA therapeutic targets for asthma only were restricted to Th2 asthma, but not Th17 asthma or severe asthma. We first demonstrated that miR-146a-3p suppressed Th17 differentiation by targeting MBD2 in severe asthma and miR-146a-3p overexpression significantly reduced airway hyperresponsiveness, airway inflammation and airway mucus secretion and inhibited the Th17 cells response, which suppressed the development of severe asthma, providing a potential novel therapeutic for Th17 predominant neutrophilic severe asthma.

The principal limitations in this study were that (a) it was a single-centre study with patients from one region and the number of Th17 severe asthma patients was insufficient; (b) airway inflammation in severe asthma patients was not assessed in this study but it could be further evaluated by measuring fractionated exhaled nitric oxide (FENO), induced sputum or alveolar lavage fluid; (c) there is no glucocorticoid therapy control group, although Th17 severe asthma is known to be corticosteroid-insensitive; and (d) the results indicated that miR-146a-3p inhibited Th17 differentiation by targeting the MBD2, but it is not clear how MBD2 affects Th17 differentiation.

## Conclusion

miR-146a-3p suppressed Th17 differentiation by targeting MBD2 to provide a potential novel therapeutic for Th17 predominant neutrophilic severe asthma.

### Supplementary Information

Below is the link to the electronic supplementary material.

Supplementary file1 (PDF 685 kb)

## Data Availability

The data used and analysed in this study are available from the corresponding author on reasonable request.

## Abbreviations

ACT: Asthma control test
BMI: Body mass index
BECs: Bronchial epithelial cells
BALF: Bronchoalveolar lavage fluid
HDM: House dust mite
ECP: Eosinophilic cationic protein
ELISA: Enzyme-linked immunosorbent assay
FEV1: Forced expiratory volume in 1 s
FVC: Forced vital capacity
IgE: Immune globulin E
IL-4: Interleukin-4
IQR: Interquartile range
LPS: Lipopolysaccharide
Mch: Methacholine
MBD2: Methyl-CpG binding domain protein 2
MiR: MicroRNA
NEU: Neutrophil granulocyte
OVA: Ovalbumin
PBMCs: Peripheral blood mononuclear cells
PFT: Pulmonary function test
PBMCs: Peripheral blood mononuclear cells
PBS: Phosphate-buffered saline
RORγt: Orphan nuclear receptor
RL: Lung resistance
Th17: T-helper cell type 17
Th2: T-helper cell type 2
