# Supplementary material for: miR-146a-3p as a potential novel therapeutic by targeting MBD2 to mediate Th17 differentiation in Th17 predominant neutrophilic severe asthma
Source: Clin Exp Med. 2023 Mar 24;23(6):2839–54. doi: 10.1007/s10238-023-01033-0 (PMC10543568; doi:10.1007/s10238-023-01033-0)
Supplement: Supplementary file 1 — Supplementary file1 (PDF 685 kb) [file 10238_2023_1033_MOESM1_ESM.pdf]

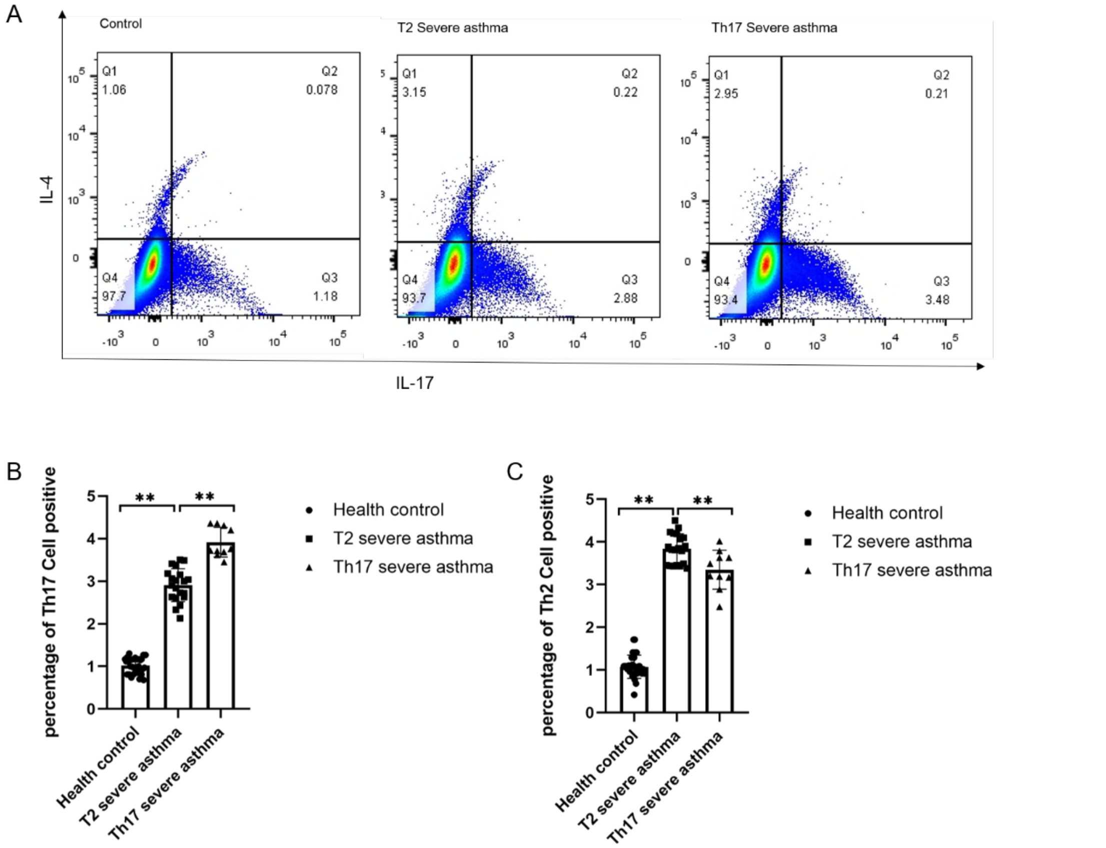

Supplementary Fig.1 Peripheral blood mononuclear cells were examined by flow cytometry to distinguish between T2 and Th17 severe asthma. A. percentage of Th17 and Th2 cells positive; B. percentage of Th17 cells positive; C. percentage of Th2 cells positive.

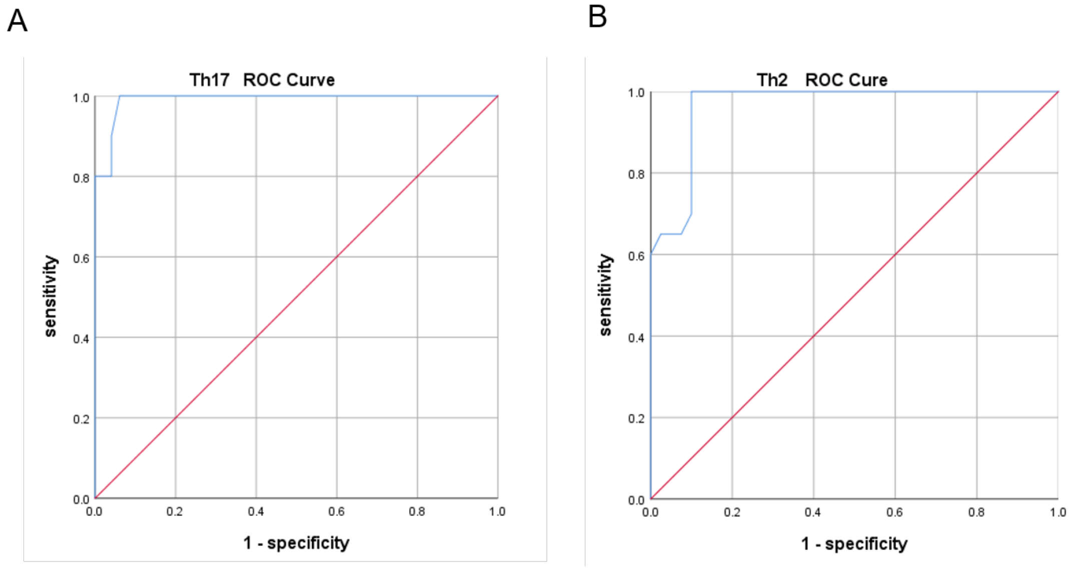

Supplementary Fig.2. Th17 and Th2 cells ROC Curve. A. Th17 ROC Curve area 95% confidence interval (0.991-1, p= 0.00), Th17 Cut off 3.095%; B. Th2 ROC Curve area 95% confidence interval (0.965-1, p= 0.00), Th2 Cut off 3.045%.

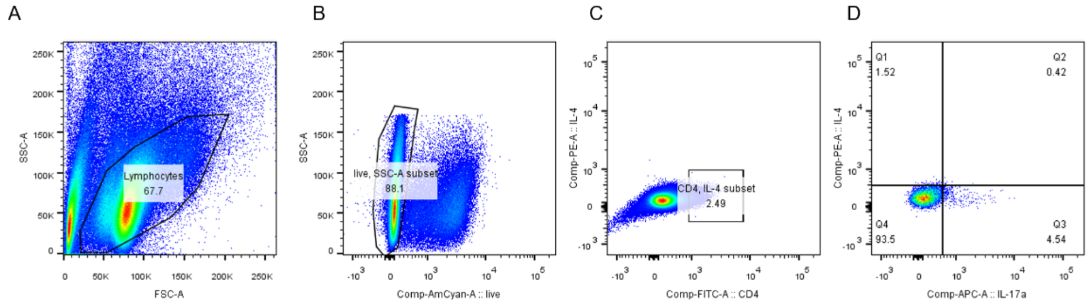

Supplementary Fig.3. Gating strategy for the identification of Th17 cells. Gating sequence, A →B →C → D. A. Gating strategy for Lymphocytes; B. Gating strategy for live cells; C. Gating strategy for $CD4^+$ cells; D. Gating strategy for the identification of IL4 and IL17.
